# Seroepidemiology of Hepatitis A in South Korea: A Nationwide Study by the Eone Reference Laboratory

**DOI:** 10.2188/jea.JE20120188

**Published:** 2013-07-05

**Authors:** Sung Eun Cho, Youngdae Kim

**Affiliations:** 1 Eone Reference Laboratory, Seoul, South Korea; 2 Department of Pediatrics, Seoul Paik Hospital, College of Medicine, Inje University, Seoul, South Korea

**Keywords:** hepatitis A virus, South Korea, immunization

## Abstract

**Background:**

We evaluated the recent prevalence of serologic markers of hepatitis A virus (HAV) in South Korea.

**Methods:**

The study data were the results of 60 126 anti-HAV (total) tests and 30 786 anti-HAV IgM tests that were performed during April 2009 through March 2010 by the Eone Reference Laboratory at the request of 1935 institutions throughout Korea.

**Results:**

The overall positivity rate was 51.06% on the anti-HAV (total) test and 11.20% on the anti-HAV IgM test. As compared with the other age groups the rate of anti-HAV (total) positivity was significantly lower (*P* < 0.001), and the rate of anti-HAV IgM positivity was significantly higher (*P* < 0.001), among Koreans aged 11 to 40 years. The seroprevalence of anti-HAV IgM significantly differed according to region but not by referral date.

**Conclusions:**

This was the largest nationwide study in South Korea by 1 laboratory, and it provides useful recent baseline data on hepatitis A in Asia. The findings suggest that active immunization of younger Koreans should be made a priority.

## INTRODUCTION

Hepatitis A is an acute, typically self-limiting liver disease and is one of the most common infectious diseases in the world. The hepatitis A virus (HAV) is a member of the Picornaviridae family of small, nonenveloped, single-stranded RNA viruses.^1^ HAV infection occurs primarily by oral inoculation of fecally excreted virus, either by person-to-person contact or by ingestion of contaminated food or water.^2^

Hepatitis A is highly endemic in developing nations with poor sanitation, where infection often occurs in children.^2^^,^^3^ In developed nations, the proportion of symptomatic patients is higher because infection is more likely in adults.^2^^,^^4^ The presence and severity of symptoms associated with HAV infection is related to patient age. Approximately 70% of infected adults develop symptoms, including jaundice. In contrast, only 30% of children younger than 6 years develop symptoms, which are usually nonspecific and flu-like, without jaundice.^2^^,^^5^

In Korea, rapid economic development has caused a rapid epidemiologic shift in HAV infection. Since the late 1990s, the number of patients with HAV infection has markedly increased in Korea, especially among those aged 20 to 40 years, most likely due to to the lower rate of anti-HAV IgG positivity in this age group.^6^^–^^13^

The rising prevalence of HAV infection has led to an increase in the number of requests for HAV serologic tests. To establish programs that are effective in preventing HAV infection, a nationwide epidemiologic study of anti-HAV seroprevalence is needed.^10^ Thus, we investigated recent nationwide epidemiologic data on anti-HAV seroprevalence in Korea in relation to sex, age group, region, and referral date.

## METHODS

The results of 60 126 anti-HAV (total) tests and 30 786 anti-HAV IgM tests that were performed during April 2009 through March 2010 by the Eone Reference Laboratory at the request of 1935 institutions throughout Korea were evaluated with respect to sex, age group, region, and referral date. The subjects were divided into 7 age groups (0–10, 11–20, 21–30, 31–40, 41–50, 51–60, >60 years). Region was classified according to the 7 provinces of South Korea, namely, (1) Seoul, (2) Gyeonggido/Incheon, (3) Gangwondo, (4) Chungcueongdo/Daejeon, (5) Gyeongsangdo/Busan/Ulsan/Daegu, (6) Jeollado/Gwangju, and (7) Jejudo. The study complied with the Declaration of Helsinki and was approved by the local ethics committee of the Eone Reference Laboratory.

Anti-HAV (total) (ie, IgM and IgG) was evaluated with an ADVIA Centaur immunoassay system (Siemens, NY, USA) and HAV total reagents (aHAVT: Siemens Healthcare Diagnostics, NY, USA), using a competitive chemiluminometric immunoassay. Anti-HAV IgM was evaluated with an ADVIA Centaur system and IgM HAV reagents (aHAVM: Siemens Healthcare Diagnostics, NY, USA), using an antibody capture microparticle chemiluminometric immunoassay. The cut-off was 20.0 IU/L for the reactivity of anti-HAV (total) and 1.2 S/Co (signal-to-cut-off) for the reactivity of anti-HAV IgM.

### Statistical analysis

Statistical analysis was performed using the SPSS program (version 18.0; SPSS Inc., Chicago, IL, USA). χ^2^ analysis was used to compare categorical variables, and χ^2^ analysis with linear-by-linear association was used to evaluate trends in seroprevalence according to age group and referral date. A *P* value less than 0.05 was considered to indicate statistical significance.

## RESULTS

The overall rate of positive test results was 51.06% for anti-HAV (total) and 11.20% for anti-HAV IgM. The rate of anti-HAV (total) positivity did not significantly differ between males and females (52.86% vs 49.44%, *P* = 0.560); however, the rate of anti-HAV IgM positivity was higher among males than among females (13.35% vs 8.82%, *P* = 0.020; Table 1).

**Table 1.** Rates of anti-HAV (total) and anti-HAV IgM positivity according to sex

	Male	Female	Total	*P*-value*
Anti-HAV (total)	52.86% (14 987/28 350)	49.44% (15 712/31 776)	51.06% (30 699/60 126)	NS
Anti-HAV IgM	13.35% (2159/16 167)	8.82% (1290/14 619)	11.20% (3449/30 786)	0.020

HAV: hepatitis A virus.

*χ^2^ analysis. NS, not significant.

As compared with the other age groups, the rate of anti-HAV (total) positivity was significantly lower (*P* < 0.001), and the rate of IgM anti-HAV positivity was significantly higher (*P* < 0.001), in the age groups encompassing individuals aged 11 to 40 years (Table 2). The rate of anti-HAV (total) positivity was 55.48% in the age group 0 to 10 years, which was higher than in the age groups that included individuals aged 11 to 40 years. The rate of anti-HAV (total) positivity was greater than 90% in the age groups 41 to 50 years, 51 to 60 years, and 61 years or older. Most importantly, there was a trend toward an inverse relation between rate of anti-HAV (total) positivity and anti-HAV IgM positivity in all age groups (Table 2).

**Table 2.** Rates of anti-HAV (total) and anti-HAV IgM positivity according to age group

Age, years	Anti-HAV (total)	Anti-HAV IgM
0–10	55.48% (542/977)	2.05% (12/585)
11–20	22.56% (1545/6849)	8.36% (258/3086)
21–30	14.20% (1985/13 976)	18.48% (1347/7287)
31–40	50.69% (9370/18 485)	15.63% (1330/8512)
41–50	91.38% (8317/9102)	6.94% (314/4524)
51–60	99.05% (4505/4548)	0.75% (21/2799)
>60	96.02% (2579/2686)	0.73% (17/2333)
Total	50.94% (28 843/56 623)	10.54% (3316/31 459)
*P*-value*	<0.001	<0.001

HAV: hepatitis A virus.

*χ^2^ analysis with linear-by-linear association. NS, not significant.

The seroprevalence of anti-HAV IgM significantly differed according to region and was higher in Seoul, Gyeonggido/Incheon, and Gangwondo (*P* = 0.023) (Table 3; Figure).

**Figure.**
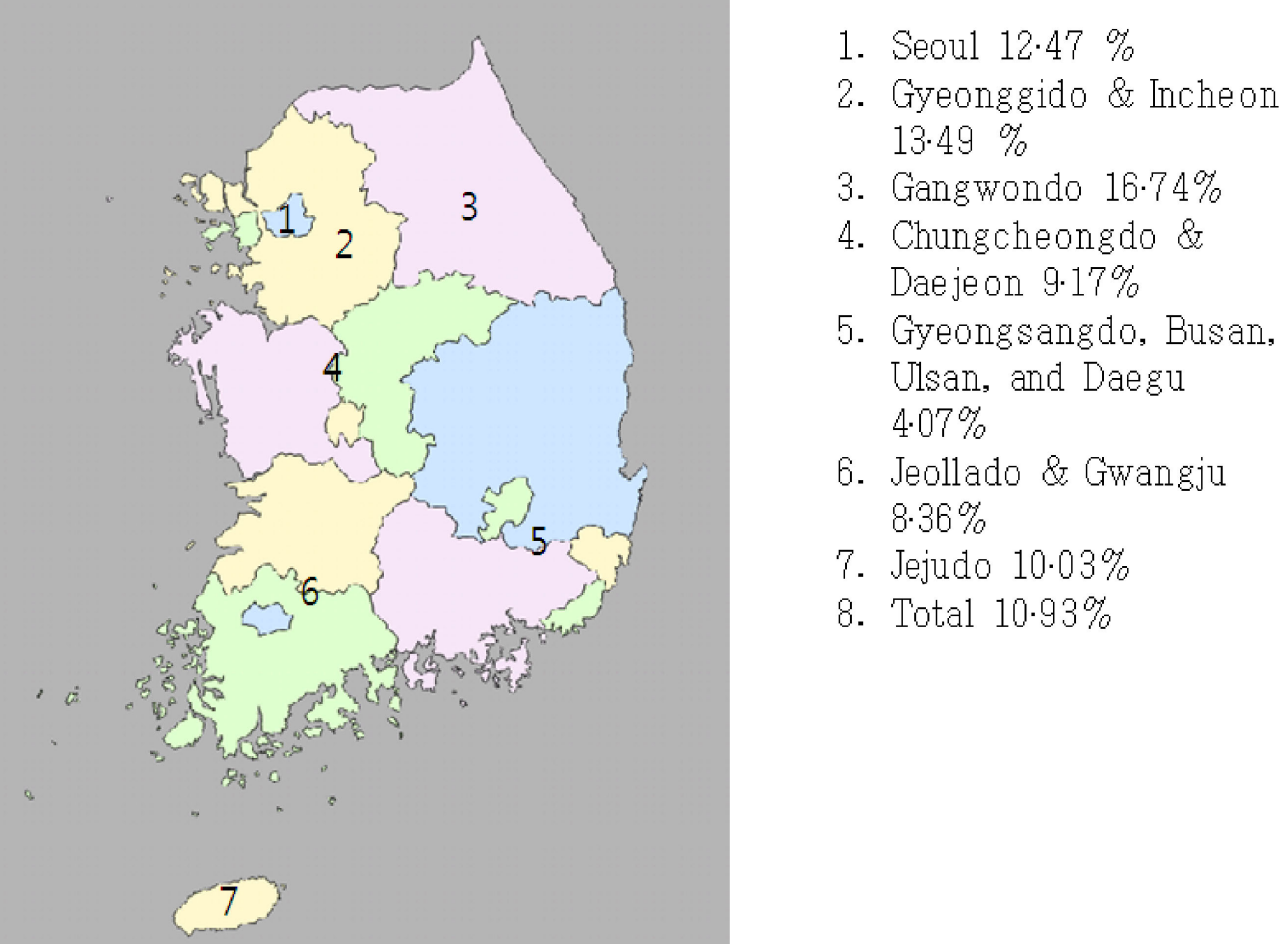
Rate of anti-HAV IgM positivity according to region (*P* = 0.023). HAV: hepatitis A virus.

**Table 3.** Rates of anti-HAV (total) and anti-HAV IgM positivity according to region

	Anti-HAV (total)	Anti-HAV IgM
Seoul	46.54% (13 767/29 580)	12.47% (1601/12 842)
Gyeonggido/Incheon	53.92% (13 632/25 280)	13.49% (1450/10 751)
Gangwondo	57.66% (1992/3455)	16.74% (540/3226)
Chungcheongdo/Daejeon	51.44% (3524/6851)	9.17% (292/3186)
Gyeongsangdo/Busan/Ulsan/Daegu	51.71% (4678/9047)	4.07% (279/6860)
Jeollado/Gwangju	56.67% (3142/5544)	8.36% (418/4998)
Jejudo	62.26% (447/718)	10.03% (36/359)
Total	51.17% (41 182/80 475)	10.93% (4616/42 222)
*P*-value*	NS	0.023

HAV: hepatitis A virus.

*χ^2^ analysis. NS, not significant.

Prevalence rates did not significantly differ according to referral date during the 1-year period from April 2009 through March 2010 (Table 4).

**Table 4.** Rates of anti-HAV (total) and anti-HAV IgM positivity according to referral date

	Anti-HAV (total)	Anti-HAV IgM
April 2009	60.40% (1167/1932)	23.16% (446/1926)
May 2009	47.64% (3834/8047)	14.15% (757/5350)
June 2009	49.69% (3953/7956)	13.40% (686/5118)
July 2009	48.51% (4751/9794)	11.17% (643/5757)
August 2009	51.16% (4339/8482)	8.13% (388/4770)
September 2009	54.18% (3316/6120)	8.04% (268/3333)
October 2009	52.54% (2506/4770)	8.45% (215/2543)
November 2009	50.25% (2011/4002)	8.87% (182/2053)
December 2009	54.39% (1995/3668)	13.66% (260/1904)
January 2010	53.26% (2049/3847)	13.69% (276/2016)
February 2010	50.40% (4417/8764)	6.61% (203/3072)
March 2010	52.27% (6844/13 093)	6.67% (292/4380)
Total	51.17% (41 182/80 475)	10.93% (4616/42 222)
*P*-value*	NS	NS

HAV: hepatitis A virus.

*χ^2^ analysis with linear-by-linear association. NS, not significant.

## DISCUSSION

Although a number of studies have examined anti-HAV seroprevalence in Korea,^6^^–^^24^ they were restricted to specific regions or age groups. We conducted a nationwide epidemiologic study of recent anti-HAV seroprevalence in Korea to establish effective measures to prevent HAV infection.

Overall seroprevalence was 51.06% for anti-HAV (total) and 11.20% for anti-HAV IgM. These values confirm those reported in previous studies of the Korean population. In those studies the positivity rates for all age groups combined were 45.7% to 62.8% for anti-HAV (total)^6^^,^^9^^,^^10^ and 11.0% for anti-HAV IgM.^10^

The positivity rate for anti-HAV IgM was higher in males than in females in this study (13.35% vs 8.82%, *P* = 0.020), which also confirms earlier findings in Korea.^10^^,^^15^ In a previous study, the male-female ratio was 1.2:1.0 during the period 1988–1998 among children living in Gyeonggido province.^15^ The rate of anti-HAV IgM positivity was higher in males than in females (11.8% vs 10.0%, *P* < 0.0001) in an epidemiologic study analyzing a recent 4-year period (2005–2008).^10^ In another study, the male predominance in prevalence was explained by the greater frequency of virus exposure among young men.^6^ However, as compared with previous studies of Koreans, we observed a marked predominance in prevalence among males. Because this is the most recent analysis of Korean data, this trend toward male predominance in HAV infection is likely to continue in the near future in Korea.

In accordance with the lower rate of anti-HAV (total) positivity, as compared with other age groups, the prevalence of acute hepatitis A infection (as indicated by the presence of anti-HAV IgM) was higher in the age groups that included individuals aged 11 to 40 years. HAV seroepidemiology in Korea is rapidly changing, and a growing number of young adults are susceptible to HAV infection.^9^ The decrease in HAV infection among young adults has resulted in fewer individuals with natural immunity and a consequent increase in the adult population at risk of contracting the disease.^16^ This is supported by several previous studies in Korea.^6^^–^^13^ In 1 study, the seroprevalence of anti-HAV (total) was very low among teenagers and those aged 20 to 29 years, higher among adults aged 30 to 39 years, and greater than 90% among older adults. Most people with HAV hepatitis were aged 20 to 39 years.^6^ In another study, the annual rate of anti-HAV (total) positivity was significantly lower among adults 21 years or older during a recent 4-year period (2005–2008), but the rate of anti-HAV IgM positivity showed an increasing trend.^10^

Interestingly, the positivity rate was lowest among those aged 20 to 29 years in the present study, which suggests an increased risk of hepatitis A outbreaks among military personnel, as was previously reported.^17^^,^^18^ The male predominance in anti-HAV IgM is additional evidence of such outbreaks. Korea has a military draft system, and men in their early 20s are required to serve in the armed services. Military personnel are considered to be at higher risk for HAV infection as compared with the civilian population.^19^ The seroprevalence of anti-HAV IgG among those aged 24 years or younger was 4.7% in a previous study of Korean military personnel.^20^ However, the HAV vaccination rate was very low among military soldiers.^19^ These findings indicate a need to strengthen requirements for military HAV vaccination programs.

Among adults aged 40 years or older, the rate of anti-HAV (total) positivity was greater than 90%, which indicates that natural immunity helps lower the seroprevalence of anti-HAV IgM. This is explained by the cohort effect, ie, the parallel shift during the past 20 years of age groups that possess the antibody,^16^ and reflects the fact that hepatitis A was once endemic in Korea.

The rate of anti-HAV (total) positivity was 55.48% among children younger than 10 years, which is the result of vaccination. Other studies have also observed an anti-HAV seroprevalence greater than 50% in the same age group.^6^^,^^16^^,^^21^ Routine childhood vaccination in Korea would aid in preventing and eradicating acute hepatitis A in the near future.

The seroprevalence of anti-HAV IgM significantly differed according to region: it was higher in Seoul, Gyeonggido/Incheon, and Gangwondo than in the other regions (*P* = 0.023). Both socioeconomic status and the ratio of residents aged 11 to 40 years are highest in Seoul and Gyeonggido/Incheon, which contributed to the higher seroprevalence of anti-HAV IgM in these regions. These findings are consistent with those of a previous study by the Korean Centers for Disease Control and Prevention (KCDC). However, another study found that anti-HAV IgM seroprevalence was not high in Gangwondo.^22^ Gangwondo has a large number of Korean military personnel, most of whom are male and in their 20s. The low seroprevalence of anti-HAV (total) among younger military personnel contributes to the higher seroprevalence of anti-HAV IgM in Gangwondo. There is also the possibility that HAV outbreaks have occurred among military personnel.

Prevalence rates did not significantly differ according to referral date, indicating no difference in the seasonality of hepatitis A, which is consistent with findings from previous studies.^22^^–^^24^

When the seroprevalence of anti-HAV (total) is high, selective vaccination according to anti-HAV serostatus is more cost-effective than universal vaccination. However, if seroprevalence is less than 45%, universal vaccination without screening is considered the best strategy.^25^ In this study, the overall seroprevalence of anti-HAV (total) was greater than 45% (51.06%). Although universal vaccination against HAV is not available in Korea, the epidemiologic shift of HAV indicates that Korea—and countries with similar issues—should promote childhood vaccination and consider catch-up vaccination for adolescents and young adults, including high-risk populations such as military personnel and individuals with chronic liver disease.^9^ This study has identified the age and regional groups at high risk.

In conclusion, we described the recent epidemiologic characteristics of HAV in Korea. We recommend prompt scheduling of both childhood vaccinations and catch-up vaccinations for adolescents and young adults, including high-risk populations. This is the largest nationwide study by 1 laboratory in Korea, and it provides useful baseline data on recent hepatitis A infection in Asia.
